# Ethnic differences in response to atypical antipsychotics in patients with schizophrenia: individual patient data meta-analysis of randomised placebo-controlled registration trials submitted to the Dutch Medicines Evaluation Board

**DOI:** 10.1192/bjo.2023.19

**Published:** 2023-03-02

**Authors:** Bram W. C. Storosum, Cedrine Steinz, Sem E. Cohen, Taina Mattila, Wim van den Brink, Kit Roes, Lieuwe de Haan, Damiaan A. J. P. Denys, Jasper B. Zantvoord

**Affiliations:** Department of Psychiatry, Amsterdam UMC, The Netherlands; and Amsterdam Neuroscience, University of Amsterdam, The Netherlands; Department of CNS Products, Medicines Evaluation Board, The Netherlands; Biostatistics Research Group, Department for Health Evidence, Radboud Institute for Health Sciences, Radboud University Medical Center, The Netherlands; Department of Psychiatry, Amsterdam UMC, The Netherlands; Amsterdam Neuroscience, University of Amsterdam, The Netherlands; and Department of Research, Arkin Institute for Mental Health, The Netherlands; Department of Psychiatry, Amsterdam UMC, The Netherlands; Amsterdam Neuroscience, University of Amsterdam, The Netherlands; and Netherlands Institute for Neuroscience, Royal Netherlands Academy of Arts and Sciences, The Netherlands

**Keywords:** Schizophrenia, ethnicity, efficacy, antipsychotics, individual participant data meta-analysis

## Abstract

**Background:**

Little is known about the effect of ethnicity on the response to antipsychotic medication in patients with schizophrenia.

**Aims:**

To determine whether ethnicity moderates the response to antipsychotic medication in patients with schizophrenia, and whether this moderation is independent of confounders.

**Method:**

We analysed 18 short-term, placebo-controlled registration trials of atypical antipsychotic medications in patients with schizophrenia (*N* = 3880). A two-step, random-effects, individual patient data meta-analysis was applied to establish the moderating effect of ethnicity (White versus Black) on symptom improvement according to the Brief Psychiatric Rating Scale (BPRS) and on response, defined as >30% BPRS reduction. These analyses were corrected for baseline severity, baseline negative symptoms, age and gender. A conventional meta-analysis was performed to determine the effect size of antipsychotic treatment for each ethnic group separately.

**Results:**

In the complete data-set, 61% of patients were White, 25.6% of patients were Black and 13.4% of patients were of other ethnicities. Ethnicity did not moderate the efficacy of antipsychotic treatment: pooled *β*-coefficient for the interaction between treatment and ethnic group was −0.582 (95% CI −2.567 to 1.412) for mean BPRS change, with an odds ratio of 0.875 (95% CI 0.510–1.499) for response. These results were not modified by confounders.

**Conclusions:**

Atypical antipsychotic medication is equally effective in both Black and White patients with schizophrenia. In registration trials, White and Black patients were overrepresented relative to other ethnic groups, limiting the generalisability of our findings.

Schizophrenia is a severe mental illness (lifetime prevalence of 0.7%), with a huge burden for patients, families and society. Antipsychotic medication is the mainstay of treatment.^1^ Several clinical and demographic factors have been shown to be negatively associated with treatment response to antipsychotics, including severity of baseline negative symptoms, younger age at onset and the duration of untreated psychosis.^2–4^ Male patients show a smaller effect size than female patients because of a lower placebo response in female patients.^5^ To date, little is known about the role of ethnicity on drug treatment in patients with schizophrenia. This is striking because research has shown ethnic differences in the efficacy of other medication. Black patients, for instance, benefit less from beta-blockers and angiotensin-converting enzyme inhibitors than White patients.^6^ There are also ethnic differences in dosage and prescribing practices.^7–10^ White patients are more likely to be offered a range of evidence-based treatments for psychosis,^11^ whereas Black patients have a higher rate of discontinuation of antipsychotic medication,^12^ are more likely to receive (long-acting) injectable antipsychotics^11^ and are more susceptible to adverse events such as weight gain and diabetes.^13–15^ This may be because of ethnic differences in pharmacodynamics or pharmacokinetic differences.^16–18^

Although ethnic differences in the efficacy of antipsychotics are conceivable through the same pharmacological differences that may explain the higher incidence of adverse events, previous studies have reported conflicting results. Two studies found no significant differences in the efficacy of antipsychotics between White and Black patients with schizophrenia,^13,19^ whereas one small study (79 White patients and 50 Black patients) showed a lower symptom reduction in White patients compared with Black patients (Positive and Negative Syndrome Scale (PANSS) score reduction of 11.4 *v*. 28.4, respectively).^20^ However, none of these studies had a placebo control arm and all were performed as *post hoc* analyses.

## Aims

The aim of the current study is to test whether ethnicity moderates the response to antipsychotic medication in patients with schizophrenia, and whether a potential moderating effect is dependent on baseline severity, baseline negative symptoms, age or gender.

## Method

### Selection of studies and participants

**Fig. 1:**
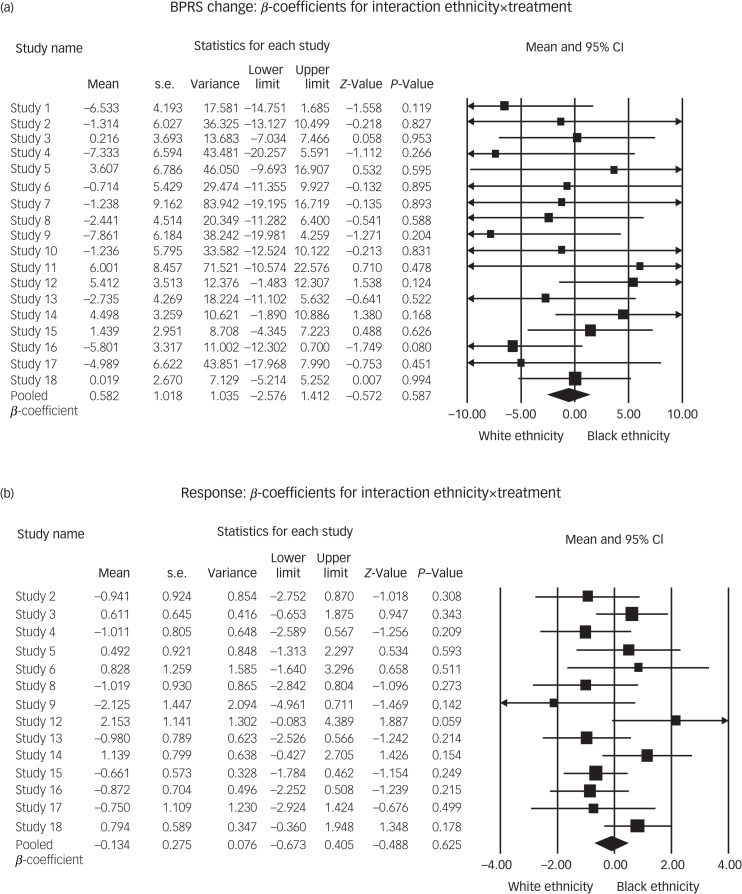
Interaction of ethnicity × treatment (with main effects) *β*-coefficients for (a) BPRS change and (b) response. BPRS, Brief Psychiatric Rating Scale.

Data were obtained from the double-blind, randomised, placebo-controlled short-term efficacy trials with antipsychotics for the treatment of psychotic episodes in patients with a DSM-III-R or DSM-IV diagnosis of schizophrenia, identified from documentation submitted by pharmaceutical companies to the Dutch regulatory authority for the purpose of marketing authorisation application. These studies (*n* = 22, including 5233 patients; 3727 on active medication and 1506 on placebo) were initiated between 1991 and 2004.^21^ A study period of 6 weeks was chosen for the analysis cut-off point because this is the duration of short-term schizophrenia trials recommended in the European Medicines Agency's Committee for Medicinal Products for Human Use (CHMP) guideline on clinical investigation of medicinal products in the treatment of schizophrenia.^22^ The Yale University Open Data Access (YODA) Project was consulted for relevant additional individual patient data from high-quality trials, which yielded no additional data (Fig. 1).

For the current study, the data were compiled with a specific focus on ethnicity. Adults (age 18 years or over) with schizophrenia receiving antipsychotic medications were included as participants and adults with schizophrenia receiving placebo were included as controls. Availability of data on ethnicity was a prerequisite for inclusion. In the database, the following predefined ethnic groups were available: Caucasian, Black, Asian, Oriental, Hispanic, Native American, other. The original individual patient studies were identified for the purpose of collecting manuscripts and corresponding authors were contacted in case of unclarities or missing information (e.g. on the definition of the ethnicity subgroups). In addition, the ethnic subgroups were examined and redefined to the terms American Indian or Alaska Native, Asian, Black, Native Hawaiian or other Pacific Islander, White and some other race, according to current JAMA Network guidelines.^23^ In the original studies, the term ‘race’ was used, or may have been used interchangeably with ‘ethnicity’. In the current report, however, only the term ethnicity is used because of the controversial implications regarding the term ‘race’ and because the concept of ethnicity encompasses a broader definition.^23^

To ensure adequately powered statistical analyses, individual studies were excluded if the ethnicity subgroups consisted of fewer than ten patients per study arm (placebo or active medication).

The five atypical antipsychotic medications that are currently most frequently prescribed in clinical practice were included. Because of agreements between the Medicines Evaluation Board and the pharmaceutical companies that provided their individual patient data, the names of the compounds cannot be disclosed in this report. To avoid bias from ineffective dosing, only studies with potential effective doses (according to current Summaries of Product Characteristics) of antipsychotic medications were included. We did not perform a formal systematic literature search, because in the design of the current study, only available individual patient data were used. Regarding background information, PubMed, EMBASE and PsycINFO were consulted. Additionally, quoted articles were checked for relevant references. To provide transparent and accurate reporting, the EQUATOR network was consulted for guidelines.^24^ To determine the risk of bias of the individual studies, the individual patient data-specific extension to the guideline for the Preferred Reporting Items for Systematic Reviews and Meta-Analysis (PRISMA) guidelines was followed. A Cochrane Risk of Bias tool was used, and the study protocol was registered in the International Prospective Register of Systematic Reviews (PROSPERO; identifier CRD42022327122).

### Outcome measures

The main efficacy measure was the Brief Psychiatric Rating Scale (BPRS). The scale consists of 18 items with potential scores ranging from 1 (not present) to 7 (extremely severe), resulting in a total score between 18 and 126. In the original individual patient data, either the BPRS or the PANSS was used for measuring symptoms. When no BPRS data were available, PANSS data were converted into BPRS scores, following the method previously described in the literature.^21^ When individual BPRS/PANNS item scores were missing, the average of the other subgroup scores of that visit were used. The main outcome measure was the difference between total BPRS scores at baseline and week 6 (defined as BPRS change). As an additional outcome measure, a minimum of 30% reduction of BPRS scores from baseline to week 6 was used (defined as response). The response criterion of 30% is in accordance with the EMA's CHMP guideline.^22^ When patients dropped out earlier than 6 weeks, the last observation was carried forward to week 6.

To examine possible confounding effects, baseline severity, baseline negative symptoms, age and gender were used as covariates in the various statistical models. To compute baseline negative symptoms, three subscale items from the BPRS were used: emotional withdrawal (item 3), motor retardation (item 13) and blunted affect (item 16), as per previous research.^21^ To provide an impression of the possible influence of the publication date of the individual studies on the results, the studies were ranked in chronological order.

### Statistical analyses

A two-step, random-effects, individual patient data meta-analysis was performed. To explore participant-level variations and to control for potential confounders, individual patient data meta-analysis was chosen over study aggregate meta-analysis. Because of existing heterogeneity between studies (e.g. different patient populations, different types of medications and different companies), random-effect instead of fixed-effect models were used.^25^

First, basic characteristics and outcome measures (BPRS change from baseline and response rates) were calculated. Subsequently, multivariate linear regression analyses were performed with mean BPRS change from baseline as the dependent variable and treatment condition, ethnicity and treatment condition×ethnicity as the independent variables. Similarly, a multivariate logistic regression analysis was performed with response as dependent variable. Thus, in both analyses, the interaction of ethnicity×treatment condition (active medication versus placebo) was added to the main effects (ethnicity and treatment condition) as independent variable as an indicator for a modifier effect of ethnicity on treatment effect. Subsequently, to examine the effect of baseline severity, baseline negative symptoms, age and gender, these variables were cumulatively added as independent variables to the main effects and the interaction of ethnicity by treatment. For these analyses, SPSS version 26 for Windows was used.

Subsequently, Comprehensive Meta-Analysis (CMA for Windows, Biostat Inc., USA; https://www.meta-analysis.com/) version 2 software was used to perform random-effect meta-analyses, with outcomes created in the abovementioned linear and logistic regression analyses, i.e. the regression coefficients and odds ratios for the treatment condition×ethnicity interactions. The 95% confidence interval indicates the scope of uncertainty in the effect estimate of the treatment condition×ethnicity interactions, considering heterogeneity between studies.

Finally, the treatment effect in the ethnicity subgroups (Black and White) was examined separately. A conventional individual patient data meta-analysis was performed, yielding an overall pooled mean difference in outcomes (BPRS change from baseline and response rates) between participants receiving antipsychotic medication and participants receiving placebo.

### Definitions

In the literature, the terms ‘race’ and ‘ethnicity’ are often used interchangeably. According to the Oxford English dictionary, early use of the word ‘race’ was applied to groups of people with obviously distinct physical characteristics such as skin colour. An influential early system dating from 1775 (*De Generis Humani Varietati Nativa* by J. F. Blumenbach) divided the human species into five races: American, Caucasian, Ethiopian, Malay and Mongolian. This system was based on conformation of the head and skin colour, and assigned the races in qualitative ranking. Because of such early theories and ideologies, the use of the word ‘race’ in reference to specific ethnic groups is avoided in the current literature. Instead, the term ‘ethnicity’ is used for human groups that entertain a subjective belief in their common descent because of similarities of physical type or of customs or both, or because of memories of colonisation and migration. This indicates that the concept of ethnicity encompasses various characteristics, such as genetic profile, culture, migration history, ethnic identity, socioeconomic factors and discrimination. In the current study, the term ‘ethnicity’ will be used rather than ‘race’, even though ‘race’ was used in certain quoted literature and in the databases. Current guidelines of describing specific ethnic groups are followed (e.g. capitalising and using adjectival forms instead of nouns): White and Black are used instead of Caucasian and African American.

## Results

### Study population

In the original database, seven different ethnic groups were described (Caucasian, Black, Asian, Oriental, Hispanic, Native American and other). Only Black and White groups had enough patients per study arm (*n* > 10) to perform statistical analyses. The ‘other’ group had patients with mixed ethnicities, rendering them unsuitable to be subdivided into the existing groups. Consequently, four of the 22 eligible studies were excluded from the primary outcome analysis (BPRS change). Of the 18 studies included for analysis, one study had a duration of 4 weeks, 13 studies had a duration of 6 weeks, one study had a duration of 7 weeks and three studies had a duration of 8 weeks. For analyses of response, an additional four studies (out of 18 studies) had to be excluded because there were not enough patients per treatment arm. Based on the Cochrane Risk of Bias Tool, all studies were determined as low risk (see Supplementary Appendix 1 available at https://doi.org/10.1192/bjo.2023.19).

Table 1 presents demographic and clinical baseline characteristics for each ethnic group. In total, 3880 patients were included: 1328 Black patients (34.2%) and 2552 White patients (65.8%). There were no relevant baseline differences between groups. For further description of individual studies, please see Supplementary Table 1.

**Table 1:** Baseline characteristics

	White			Black			Total
	Active compound	Placebo	Total	Active compound	Placebo	Total	
*n* (%)	1818 (46.9)	734 (18.9)	2552 (65.8)	940 (24.2)	388 (10.0)	1328 (34.2)	3880 (100)
Age in years, mean (s.d.)	38.5 (10.3)	38.2 (10.1)	38.4 (10.2)	38.8 (9.5)	39.2 (10.1)	38.9 (9.7)	38.6 (10.1)
Gender, % female	25.5	22.6	24.7	23.3	20.6	22.5	23.9
Baseline BPRS score, mean (s.d.)	55.3 (10.4)	55.0 (10.0)	55.2 (10.3)	54.0 (9.4)	53.0 (8.9)	53.7 (9.2)	54.7 (10.0)
Baseline BPRS negative, mean (s.d.)	9.7 (3.2)	9.5 (3.1)	9.6 (3.2)	9.5 (3.0)	9.2 (3.0)	9.4 (3.0)	9.6 (3.1)

BPRS, Brief Psychiatric Rating Scale.

### Effect size difference for antipsychotic treatment and ethnicity

Figure 1 presents the results of the individual patient data meta-analysis of the interaction ethnicity×treatment (without adjustment for confounders). The results show no significant overall treatment condition×ethnicity effect, which indicates that ethnicity does not moderate the efficacy of antipsychotic medication. This is represented by an overall pooled *β*-coefficient of −0.582 (95% CI −2.567 to 1.412) for mean BPRS change (Fig. 1(a)) (heterogeneity: *Q* = 14.71, d.f. = 17, *I*^2^ = 0.00%, *τ*^2^ = 0.00) and an overall pooled *β*-coefficient of −0.134 (95% CI −0.673 to 0.405) for response (Fig. 1(b)) (heterogeneity: *Q* = 19 565, *I*^2^ = 34%, *τ*^2^ = 0.34), with the latter translating to an odds ratio of 0.875 (95% CI 0.510–1.499). Addition of confounders to the model produced similar results. Crude pooled data on outcome measures for both ethnicities can be found in Supplementary Appendix 2. An overview of the individual mean *β*-coefficients, odds ratios and figures of the effect size differences of the cumulatively added confounders are displayed in Supplementary Appendix 3.

### Conventional meta-analyses for the two ethnicity groups

**Fig. 2:**
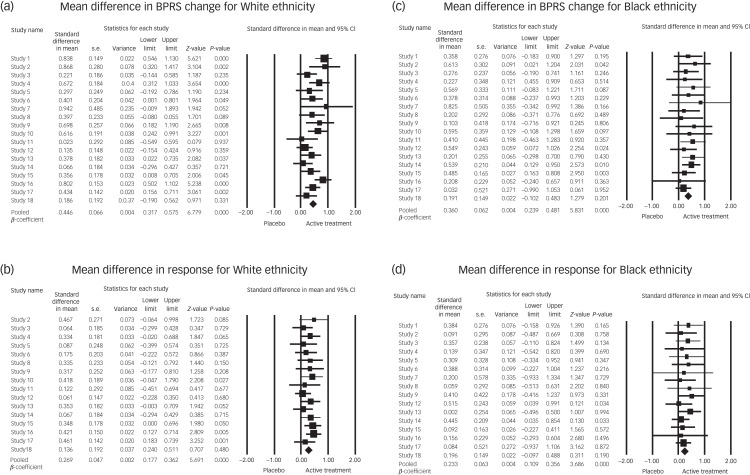
Mean difference in outcome of treatment versus placebo, according to ethnicity: (a) BPRS change for White patients, (b) response for White patients, (c) BPRS change for Black patients and (d) response for Black patients. BPRS, Brief Psychiatric Rating Scale.

Figure 2 shows the overall pooled effect sizes of BPRS change and response separately for Black and White patients. The effect sizes show a statistically significant beneficial effect of active treatment compared with placebo in both White and Black patients. In White patients, this is represented by an overall pooled mean difference of 0.446 (95% CI 0.317–0.575) for mean BPRS change (Fig. 2(a)) (heterogeneity: *Q* = 33.746, *I*^2^ = 49.62%, *τ*^2^ = 0.04), and an overall pooled odds ratio of 0.269 (95% CI 0.177–0.362) for response (Fig. 2(b)) (heterogeneity: *Q* = 10.587, *I*^2^ = 0.00%, *τ*^2^ = 0.00). In Black patients, this is represented by an overall pooled mean difference of 0.360 (95% CI 0.293–0.481) for BPRS change (Fig. 2(c)) (heterogeneity: *Q* = 7511, *I*2 = 0.00%, *τ*^2^ = 0.00), and overall pooled odds ratio of 0.233 (95% CI 0.109–0.356) for response (Fig. 2(d)) (heterogeneity: *Q* = 5915, *I*^2^ = 0.00%, *τ*^2^ = 0.00).

## Discussion

### Main findings

In these individual patient data meta-analyses we did not find a significant effect of ethnicity in the efficacy of antipsychotics for the treatment of White and Black patients with schizophrenia for symptom improvement (BPRS change score) as well as for response. This finding was independent of baseline severity, baseline negative symptoms, age and gender. Furthermore, our results showed that antipsychotics were effective (separation between active compound and placebo) in both Black and White patients separately.

Our data-set included more White patients than Black patients (65.8% White patients compared with 34.2% Black patients) (Table 1). In the complete data-set without the exclusion of non-relevant studies, 61% of patients were White, 25.6% of patients were Black and only 13.4% of patients were Asian, Oriental, Hispanic, Native American or defined as other ethnicity, indicating that White and Black patients were overrepresented in registration trials when compared with the ethnic distribution of the population of the USA. These findings are in line with results from previous trials showing that minorities are underrepresented in clinical trials.^26^ The uneven distribution of ethnicity in our large sample of patients with schizophrenia supports the need to include more ethnic diverse populations in future clinical trials, to better represent the clinical population.

To the best of our knowledge, our study is the first to investigate the efficacy of antipsychotics in different ethnicity groups in a large sample of placebo-controlled trials with a predefined protocol.

One possible explanation for our negative findings is that ethnicity encompasses a broad definition including many factors. When investigating ethnic differences, it is not possible in general to control for all factors that may influence differences between ethnic groups. Moreover, although there are some indications that there is a difference in side-effects between different ethnic groups and that genetic ancestry could influence dopamine receptor availability, a review of 51 studies describing side-effects found limited evidence of ethnic differences in the risk of adverse events.^13–16^ In addition, genetic profiles vary widely within ethnic groups and there is no proof of an underlying explanation for possible differences in the prevalence of side-effects.

Another possible explanation for the lack of ethnic efficacy differences in the current study is that there are clinical disparities in diagnosing schizophrenia in different ethnic groups. More specifically, there is evidence that psychiatrists tend to over-diagnose schizophrenia in Black patients compared with White patients, despite the actual prevalence of schizophrenia being equally distributed over different ethnicities.^10,27^ Overdiagnosis of schizophrenia in Black patients may result in a smaller observable treatment effect of antipsychotic medication and/or a higher placebo response.

### Strengths and limitations

The main strength of our study is the inclusion of a large number of individual patient data. All studies included were double-blind, randomised placebo-controlled trials. This increased the reliability and generalisability of our findings, by quantifying the effect modification and accounting for heterogeneity between studies.

However, the study also has limitations. Not all provided studies could be included, which could limit the generalisability of our findings. Second, because the enrolment of included studies was between 1991 and 2004, the newest antipsychotic medications were not examined. However, medications included in the current study are still the most prescribed antipsychotics in current clinical practice.^28^ In addition, because of agreements with pharmaceutical companies, we were not able to examine the different antipsychotics individually. This may be an important limitation since in antihypertensives, there is an inter-ethnic variance of effectiveness between different medication classes.^29^ The fact that we could not examine the antipsychotics individually may mask possible ethnic differences for specific antipsychotic medications.

Although six different confounders were investigated, not all information about possible relevant confounders was available in the data-set. For example, the duration of untreated psychosis and the age at onset of the disorder are negatively correlated with treatment outcome in schizophrenia ^2,3^ and these data were not available in our data-set. Moreover, there was no information about the degree of therapy adherence, which has previously been shown to be lower in Black patients and could have resulted in a smaller treatment effect.^12^ Because of a lack of complete information on the inclusion date of patients, we were not able to examine the inclusion date as a confounder to the model. However, the forest plots were sorted based on publication date of the studies (with ascending date ranging from 1990 to 2004). When viewing these forest plots, there does not seem to be an influence of publication date on the moderating effect of ethnicity.

### Implications

Our findings confirm the presence of equal efficacy of antipsychotic medications in both White and Black patients with schizophrenia. Our findings also show that different ethnicities are not evenly distributed in large clinical trials, emphasising the need for more diversity in research, to ensure a more representative distribution of ethnic groups.

## Data Availability

Restrictions apply to the availability of the data that support the findings of this study. Data are available from the authors with permission from the pharmaceutical companies.

## Supplementary material

For supplementary material accompanying this paper visit http://doi.org/10.1192/bjo.2023.19. click here to view supplementary material
