# Supplementary material for: Ethnic differences in response to atypical antipsychotics in patients with schizophrenia: individual patient data meta-analysis of randomised placebo-controlled registration trials submitted to the Dutch Medicines Evaluation Board
Source: BJPsych Open. 2023 Mar 2;9(2):e45. doi: 10.1192/bjo.2023.19 (PMC10044330; doi:10.1192/bjo.2023.19)
Supplement: Supplementary file 1 [file S2056472423000194sup001.docx]

| **Study no.** | **Study performed in** | **Study start year** | **Drug** | **Patients exp/cont N** | **Age mean (SD)** | **Sex (% male)** | **Ethnicity (%)** | **Duration of study (weeks)** | **BPRS score at baseline mean (SD)** |
| --- | --- | --- | --- | --- | --- | --- | --- | --- | --- |
| **Patient characteristics per study** | | | | | | | | | |
| **1** | North America | 1991 | A | 127/65 | 35.9 (9.46) | 84.90 | Caucasian 74 | 6 | 59.88 (10.72) |
|  |  |  |  |  |  |  | Black 18.8 |  |  |
|  |  |  |  |  |  |  | Hispanic 5.7 |  |  |
|  |  |  |  |  |  |  | Other 1.6 |  |  |
| **2** | North America | 1993 | A | 49/48 | 37.7 (9.48) | 70.10 | Caucasian 66 | 6 | 55.05 (7.71) |
|  |  |  |  |  |  |  | Black 23.7 |  |  |
|  |  |  |  |  |  |  | Hispanic 5.2 |  |  |
|  |  |  |  |  |  |  | Other 3.1 |  |  |
| **3** | Europe | 2004 | B | 75/37 | 69.7 (4.49) | 27.70 | Caucasian 100 | 6 | 50.72 (5.73) |
| **4** | Europe, Rest | 2004 | B | 374/127 | 37.3 (10.8) | 53.50 | Caucasian 85.6 | 6 | 52.36 (6.68) |
|  |  |  |  |  |  |  | Other 14.2 |  |  |
| **5** | North America | 2004 | B | 224/105 | 41.9 (10.5) | 72.00 | Caucasian 42.6 | 6 | 54.03 (7.14) |
|  |  |  |  |  |  |  | Black 56.2 |  |  |
|  |  |  |  |  |  |  | Other 1.2 |  |  |
| **6** | North America, Europe, Rest | 2004 | B | 358/120 | 36.9 (10.7) | 65.30 | Caucasian 49.6 | 6 | 52.4 (7.62) |
|  |  |  |  |  |  |  | Black 20.7 |  |  |
|  |  |  |  |  |  |  | Asian 23.9 |  |  |
|  |  |  |  |  |  |  | Other 5.9 |  |  |
| **7** | North America | 1989 | C | 8/4 | 34.1 (9.3) | 100 | Caucasian 25.0 | 3 | 41.67 (9.55) |
|  |  |  |  |  |  |  | Black 66.7 |  |  |
|  |  |  |  |  |  |  | Hispanic 8.3 |  |  |
| **8** | North America | 1991 | C | 53/53 | 36.3 (8.4) | 90.60 | Caucasian 61.3 | 6 | 54.94 (7.72) |
|  |  |  |  |  |  |  | Black 35.9 |  |  |
|  |  |  |  |  |  |  | Hispanic 0.9 |  |  |
|  |  |  |  |  |  |  | Other 1.9 |  |  |
| **9** | North America | 1993 | C | 203/51 | 37.1 (9.1) | 75.20 | Caucasian 71.3 | 6 | 63.37 (10.51) |
|  |  |  |  |  |  |  | Black 19.7 |  |  |
|  |  |  |  |  |  |  | Asian 2 |  |  |
|  |  |  |  |  |  |  | Hispanic 5.1 |  |  |
|  |  |  |  |  |  |  | Other 2 |  |  |
| **10** | North America | 2001 | C | 266/82 | 38.7 (10.2) | 75.60 | Caucasian 51.7 | 6 | 52.87 (9.53) |
|  |  |  |  |  |  |  | Black 36.2 |  |  |
|  |  |  |  |  |  |  | Asian 1.2 |  |  |
|  |  |  |  |  |  |  | Hispanic 9.5 |  |  |
|  |  |  |  |  |  |  | Other 1.5 |  |  |
| **11** | North America | 1991 | C | 110/57 | 37.2 (7.8) | 77.30 | Caucasian 61.7 | 6 | 56.35 (8.80) |
|  |  |  |  |  |  |  | Black 28.7 |  |  |
|  |  |  |  |  |  |  | Hispanic 9 |  |  |
|  |  |  |  |  |  |  | Other 0.6 |  |  |
| **12** | Europe | 1991 | C | 76/36 | 36.8 (11.0) | 62.50 | Caucasian 84.8 | 6 | 58.96 (10.82) |
|  |  |  |  |  |  |  | Black 4.5 |  |  |
|  |  |  |  |  |  |  | Asian 3.6 |  |  |
|  |  |  |  |  |  |  | Oriental 0.9 |  |  |
|  |  |  |  |  |  |  | Other 6.3 |  |  |
| **13** | Europe, Rest | 2004 | C | 339/115 | 34.2 (10.5) | 60.80 | Caucasian 59 | 6 | 54.03 (8.00) |
|  |  |  |  |  |  |  | Black 4.2 |  |  |
|  |  |  |  |  |  |  | Oriental 36.6 |  |  |
|  |  |  |  |  |  |  | Other 0.2 |  |  |
| **14** | North America | 2004 | C | 324/111 | 41.5 (10.2) | 73.60 | Caucasian 34.0 | 6 | 53.59 (7.52) |
|  |  |  |  |  |  |  | Black 57.5 |  |  |
|  |  |  |  |  |  |  | Asian 0.7 |  |  |
|  |  |  |  |  |  |  | Oriental 1.4 |  |  |
|  |  |  |  |  |  |  | Hispanic 4.1 |  |  |
|  |  |  |  |  |  |  | Other 2.3 |  |  |
| **15** | North America | 1990^[a](https://www.sciencedirect.com/science/article/pii/S0924977X14000637?via%3Dihub#tbl1fna)^ | D | 340/86 | 37.2 (10.5) | 83.30 | Caucasian 71.1 | 8 | 52.41 (11.07) |
|  |  |  |  |  |  |  | Black 18.1 |  |  |
|  |  |  |  |  |  |  | Oriental 2.1 |  |  |
|  |  |  |  |  |  |  | Hispanic 8.5 |  |  |
|  |  |  |  |  |  |  | Other 0.2 |  |  |
| **16** | North America | 1990[^a^](https://www.sciencedirect.com/science/article/pii/S0924977X14000637?via%3Dihub#tbl1fna) | D | 51/53 | 40.2 (10.3) | 97.10 | Caucasian 53.8 | 6 | 54.44 (11.87) |
|  |  |  |  |  |  |  | Black 44.2 |  |  |
|  |  |  |  |  |  |  | Oriental 1.0 |  |  |
|  |  |  |  |  |  |  | Hispanic 1.0 |  |  |
|  |  |  |  |  |  |  | Other 1.5 |  |  |
| **17** | North America | 1990 | D | 157/79 | 38.1 (9.6) | 79.70 | Caucasian 55.1 | 4 | 53.82 (6.43) |
|  |  |  |  |  |  |  | Black 33.9 |  |  |
|  |  |  |  |  |  |  | Asian 0.4 |  |  |
|  |  |  |  |  |  |  | Oriental 0.9 |  |  |
|  |  |  |  |  |  |  | Hispanic 9.3 |  |  |
|  |  |  |  |  |  |  | Other 0.4 |  |  |
| **18** | North America | 1992 | E | 26/11 | 33.9 (7.4) | 91.90 | Caucasian 56.7 | 7 | 52.24 (9.54) |
|  |  |  |  |  |  |  | Black 43.2 |  |  |
| **19** | North America | 1992 | E | 103/47 | 37.4 (8.6) | 96 | Caucasian 56 | 6 | 51.12 (9.87) |
|  |  |  |  |  |  |  | Black 36.7 |  |  |
|  |  |  |  |  |  |  | Asian 0.7 |  |  |
|  |  |  |  |  |  |  | Oriental 0.7 |  |  |
|  |  |  |  |  |  |  | Hispanic 5.3 |  |  |
|  |  |  |  |  |  |  | Other 0.7 |  |  |
| **20** | North America | 1993 | E | 34/39 | 40.2 (8.6) | 97.30 | Caucasian 64.4 | 6 | 53.01 (12.17) |
|  |  |  |  |  |  |  | Black 28.8 |  |  |
|  |  |  |  |  |  |  | Oriental 1.4 |  |  |
|  |  |  |  |  |  |  | Hispanic 5.9 |  |  |
| **21** | North America | 1994 | E | 222/108 | 38 (9.1) | 76.40 | Caucasian 64.9 | 8 | 52.37 (11.67) |
|  |  |  |  |  |  |  | Black 23.9 |  |  |
|  |  |  |  |  |  |  | Asian 0.6 |  |  |
|  |  |  |  |  |  |  | Oriental 2.7 |  |  |
|  |  |  |  |  |  |  | Hispanic 5.8 |  |  |
|  |  |  |  |  |  |  | Other 2.1 |  |  |
| **22** | North America | 1994 | E | 207/71 | 39.1 (9.6) | 75.90 | Caucasian 61.5 | 8 | 54.21 (13.34) |
|  |  |  |  |  |  |  | Black 28.8 |  |  |
|  |  |  |  |  |  |  | Asian 0.4 |  |  |
|  |  |  |  |  |  |  | Oriental 0.4 |  |  |
|  |  |  |  |  |  |  | Hispanic 8.3 |  |  |
|  |  |  |  |  |  |  | Other 0.7 |  |  |
|  | | | | | | | | | |
| **Patient characteristics per geographical area** | | | | | |  |  |  |  |
|  | **North America** | 1989–2004 | All | 2625/1112 | 38.6 (9.9) | 78.9 | Caucasian 56.7 | 3–8 | 54.2 (9.8) |
|  |  |  |  |  |  |  | Black 34.7 |  |  |
|  |  |  |  |  |  |  | Asian 0.5 |  |  |
|  |  |  |  |  |  |  | Oriental 0.9 |  |  |
|  |  |  |  |  |  |  | Hispanic 5.6 |  |  |
|  |  |  |  |  |  |  | Other 1.6 |  |  |
|  | **Europe** | 1991–2004 | B, C | 770/282 | 40.9 (14.5) | 50.8 | Caucasian 98.0 | 6 | 53.3 (7.7) |
|  |  |  |  |  |  |  | Black 0.7 |  |  |
|  |  |  |  |  |  |  | Asian 0.6 |  |  |
|  |  |  |  |  |  |  | Oriental 0.1 |  |  |
|  |  |  |  |  |  |  | Other 0.7 |  |  |
|  | **Rest of the world (Asia, Africa)** | 2004 | B, C | 332/112 | 32.8 (9.5) | 69.8 | Caucasian 9.7 | 6 | 52.2 (8.1) |
|  |  |  |  |  |  |  | Black 8.8 |  |  |
|  |  |  |  |  |  |  | Asian 24.8 |  |  |
|  |  |  |  |  |  |  | Oriental 37.4 |  |  |
|  |  |  |  |  |  |  | Other 19.4 |  |  |
|  | **Total** | 1989–2004 | All | 3727/1506 | 38.6 (11.1) | 72.5 | Caucasian 60.9 | 3–8 | 54.0 (9.6) |
|  |  |  |  |  |  |  | Black 25.8 |  |  |
|  |  |  |  |  |  |  | Asian 2.6 |  |  |
|  |  |  |  |  |  |  | Oriental 3.8 |  |  |
|  |  |  |  |  |  |  | Hispanic 4.0 |  |  |
|  |  |  |  |  |  |  | Other 2.9 |  |  |

a

Exact study year is unknown.
